# Comparison of Two Partially Covered Metal Stents for Endoscopic Ultrasound‐guided Hepaticogastrostomy: A Multi‐Center Retrospective Study

**DOI:** 10.1002/deo2.70396

**Published:** 2026-09-10

**Authors:** Masahiro Yamamura, Hirotoshi Ishiwatari, Saburo Matsubara, Takeshi Okamoto, Michihiro Ono, Toshinori Okuda, Etsuji Ishida, Kohichi Takada, Kazuma Ishikawa, Naoki Sasahira, Kentaro Suda, Morito Ikeda, Daichi Watanabe, Junya Sato, Hiroki Sakamoto, Akihiro Ohba, Takuya Doi, Akifumi Notsu

**Affiliations:** ^1^ Division of Pancreatobiliary Medicine Shizuoka Cancer Center Nagaizumi Japan; ^2^ Department of Gastroenterology and Hepatology Saitama Medical Center Saitama Medical University Kawagoe Japan; ^3^ Department of Hepato‐Biliary‐Pancreatic Medicine Cancer Institute Hospital of Japanese Foundation For Cancer Research Tokyo Japan; ^4^ Department of Pancreatobiliary Medicine Steel Memorial Muroran Hospital Muroran Japan; ^5^ Department of Gastroenterology Oji General Hospital Tomakomai Japan; ^6^ Department of Gastroenterology Kurashiki Central Hospital Kurashiki Japan; ^7^ Department of Medical Oncology Sapporo Medical University Sapporo Japan; ^8^ Clinical Research Center Shizuoka Cancer Center Nagaizumi Japan

**Keywords:** adverse events, axial force, endoscopic ultrasound‐guided hepaticogastrostomy, metallic stent properties, stent migration

## Abstract

**Objectives:**

Endoscopic ultrasound‐guided hepaticogastrostomy (EUS‐HGS) is an alternative biliary drainage method after failed endoscopic retrograde cholangiopancreatography. Nevertheless, it is associated with adverse events (AEs), including peritonitis and stent migration. The influence of metallic stent (MS) design characteristics, including axial force (AF), on the safety and efficacy of EUS‐HGS remains unclear.

**Methods:**

This multicenter retrospective study included patients with unresectable malignant biliary obstruction who underwent EUS‐HGS using either a high‐AF MS (Niti‐S biliary S‐type) or a low‐AF MS (EGIS biliary) between January 2019 and December 2022. Propensity score matching was performed in a 1:2 ratio. Outcomes included clinical success, early and late non‐recurrent biliary obstruction (non‐RBO) AEs, RBO, time to RBO (TRBO), and overall survival (OS).

**Results:**

After matching, 27 patients in the high‐AF group and 54 in the low‐AF group were analyzed. Clinical success, RBO rate, TRBO, OS, and late non‐RBO AE rates were similar between groups. However, early non‐RBO AEs were more frequent in the high‐AF group (37% vs. 13%, *p* **=** 0.02).

**Conclusions:**

Of the two partially covered MSs evaluated, the EGIS biliary stent demonstrated fewer early non‐RBO AEs after EUS‐HGS.

## 1 Introduction

The standard biliary drainage procedure for malignant biliary obstruction is endoscopic retrograde cholangiopancreatography (ERCP) [1, 2, 3, 4, 5]. However, ERCP can fail because of unsuccessful bile duct cannulation, duodenal obstruction, or surgically altered anatomy. In such cases, endoscopic ultrasound‐guided hepaticogastrostomy (EUS‐HGS) and EUS‐guided choledochoduodenostomy are potential alternatives [6, 7, 8, 9, 10, 11]. EUS‐HGS is often the only feasible option for duodenal obstruction or surgically altered anatomy because of limited access to the biliary system from the duodenum. Nevertheless, EUS‐HGS carries a risk of adverse events (AEs), including peritonitis, bile leakage, and stent migration, with incidences of 15.5%–29% [6, 7, 12, 13]. Current guidelines recommend covered metallic stent (MS) use in EUS‐HGS [7, 14]. Few studies have examined the effects of MS characteristics on EUS‐HGS outcomes. One key property is axial force (AF), proposed by Isayama et al., defined as the force that straightens the stent when bent. In transpapillary MS placement, high‐AF stents are associated with higher AE rates, such as pancreatitis and cholecystitis, because of greater compression at the pancreatic duct or cystic duct orifice [15, 16, 17, 18, 19]. In EUS‐HGS, high‐AF stents generate linear tension, potentially deviating the bile duct axis or compressing the bile duct wall. The more conformable low‐AF stents may provide a better fit and reduce AEs. MSs are classified by manufacturing process as laser‐cut, cross, or hook‐and‐cross types [20]. Cross‐type stents have high AF, whereas hook‐and‐cross‐type stents have low AF [20]. Therefore, our study compared the efficacy and safety of EUS‐HGS using cross‐type versus hook‐and‐cross‐type MSs.

## 2 Methods

### 2.1 Study Design

This multicenter retrospective study was conducted at seven institutions. Data from consecutive patients diagnosed with unresectable malignant biliary obstruction between January 2019 and December 2022 were retrieved from electronic databases. We included patients with malignant biliary obstruction who underwent EUS‐HGS using one of two MSs: the Niti‐S biliary S‐type (Taewoong Medical, Seoul, Korea) or EGIS biliary (S&G Biotech, Seoul, Korea). The former is a high‐AF, cross‐type MS, and the latter is a low‐AF, hook‐and‐cross‐type MS [20]. Exclusion criteria included prior biliary drainage procedures, such as percutaneous transhepatic biliary drainage, endoscopic nasobiliary drainage, deployment of metal or plastic stents via ERCP, or EUS‐HGS using a plastic stent.

### 2.2 Evaluated MSs

**FIGURE 1:**
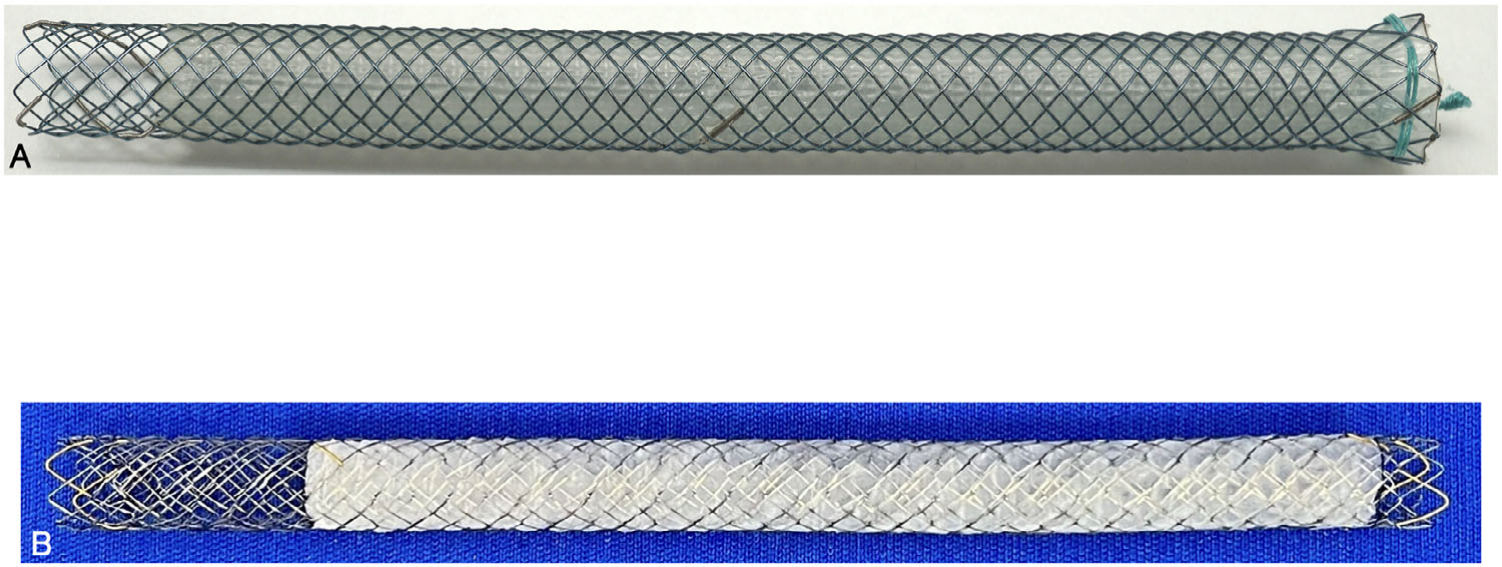
(A) Partially covered self‐expandable metallic stent (Niti‐S biliary S‐type; Taewoong Medical, Seoul, Korea). The hepatic end has a 1‐cm uncovered portion, and the gastric end has a flared portion. (B) Double‐bare, partially covered self‐expandable metallic stent (EGIS biliary; S&G Biotech, Seoul, Korea). The hepatic end has a 2‐cm uncovered portion.

The Niti‐S biliary S‐type consists of braided nitinol wire and is partially covered with a silicone membrane. The hepatic end has a 1‐cm uncovered portion, and the gastric end has a flared portion (Figure 1). We used stents that were 6 or 8 mm in diameter and 8, 10, or 12 cm in length, with an 8.5‐Fr delivery system. Stent length and diameter were selected at the discretion of the endoscopist. The EGIS biliary stent has a hook‐and‐cross braided nitinol structure with a unique double‐layer design, resulting in dense and numerous cells. The hepatic end has a 2‐cm uncovered portion. We used stents measuring 8 mm in diameter and 12 cm in length, with an 8.0‐Fr delivery system (Figure 1).

### 2.3 Endoscopic Ultrasound‐guided HGS

EUS‐HGS procedures were performed by expert endoscopists or under their direct supervision. The experts had experience with more than 30 EUS‐HGS procedures and other interventional EUS techniques, including biliary and pancreatic drainage. A curved linear‐array echoendoscope was inserted into the stomach. After excluding intervening vessels using color Doppler imaging, the intrahepatic bile duct in segment 2 or 3 (B2 or B3) was punctured using a 19‐ or 22‐gauge needle. A 0.025‐ or 0.018‐inch guidewire was then advanced into the bile duct and coiled. After tract dilation, an MS (Niti‐S biliary S‐type or EGIS biliary) was advanced and deployed between the intrahepatic duct and the stomach. In some cases, antegrade stenting (AS) was performed before HGS stent placement at the discretion of the endoscopist. After insertion of an ERCP catheter over the guidewire, the guidewire was advanced beyond the biliary obstruction and the major duodenal papilla and coiled in the duodenum. Following contrast injection to confirm the location of the obstruction and papilla, an MS was placed across the obstruction, either above or across the papilla. Representative dilation devices included a 7‐Fr bougie dilator (ES dilator; Zeon Medical, Tokyo, Japan), a 4‐mm balloon catheter (REN Biliary Balloon Dilation Catheter; Kaneka, Osaka, Japan), and a 6‐Fr cystotome (Cysto‐Gastro‐Set; Endo‐Flex GmbH, Voerde, Germany). The choice of MS and dilation device was at the endoscopist's discretion.

### 2.4 Outcome Measures

The variables compared between the Niti‐S and EGIS groups, defined according to the Tokyo Criteria 2024, were: clinical success of EUS‐HGS, recurrent biliary obstruction (RBO), causes of RBO, time to RBO (TRBO), early and late non‐RBO AEs, and overall survival (OS). Technical success was defined as successful deployment of the MS in the intended location between the intrahepatic bile duct and the stomach. Clinical success was defined as a decrease in bilirubin levels to normal or to <50% of the pretreatment value within 14 days after EUS‐HGS [21]. AEs included both RBO and non‐RBO events. RBO was defined as stent occlusion or migration resulting in recurrent biliary obstruction or requiring biliary drainage or stent removal after achieving technical and clinical success [21]. Stent migration was defined as radiologic or endoscopic displacement of the stent that required additional intervention, regardless of whether RBO had occurred. TRBO was defined as the time from EUS‐HGS to the occurrence of RBO [21]. OS was measured from the day of EUS‐HGS to patient death [21]. Non‐RBO AEs were evaluated according to the severity grading system of the American Society for Gastrointestinal Endoscopy lexicon [22]. Non‐RBO AEs were classified as early if occurring within 14 days after EUS‐HGS and as late if occurring more than 14 days after the procedure. Peritonitis was diagnosed based on clinical signs of peritoneal inflammation and corresponding fluid collection on CT [23, 24, 25].

### 2.5 Statistical Analysis

Propensity score matching at a 1:2 ratio was used to reduce imbalance and selection bias between the Niti‐S and EGIS groups. Variables previously reported as associated with AEs were considered potential confounders, including history of cholecystectomy, performance status, presence of ascites, and use of AS. Matching was performed using a 0.2‐caliper width based on the standard deviation of the logit‐transformed scores. Balance of baseline characteristics between the groups was assessed using standardized mean differences, with values <0.25 indicating adequate balance. Continuous variables are presented as medians and interquartile ranges and were analyzed using the Mann–Whitney U test. Categorical variables are presented as proportions and were analyzed using Fisher's exact test. TRBO and OS were estimated using the Kaplan–Meier method, and differences between groups were compared using the log‐rank test. To estimate TRBO, patient death and non‐RBO AEs requiring MS removal were treated as censored events at the time of death and MS removal, respectively [21]. The TRBO censoring date was defined as the date of death or the last follow‐up in patients without RBO, and the date of stent removal for reasons other than RBO. For OS analysis, the censoring date was defined as the last follow‐up for patients who were alive. The final follow‐up date was December 31, 2023. Statistical significance was set at *p* < 0.05. All statistical analyses were performed using EZR (Saitama Medical Center, Jichi Medical University, Saitama, Japan), a graphical user interface for R (R Foundation for Statistical Computing, Vienna, Austria) designed for biostatistical analyses. Additionally, Cox proportional hazards regression analysis using the entire cohort was performed to identify factors associated with TRBO.

## 3 Results

### 3.1 Patient Characteristics

**FIGURE 2:**
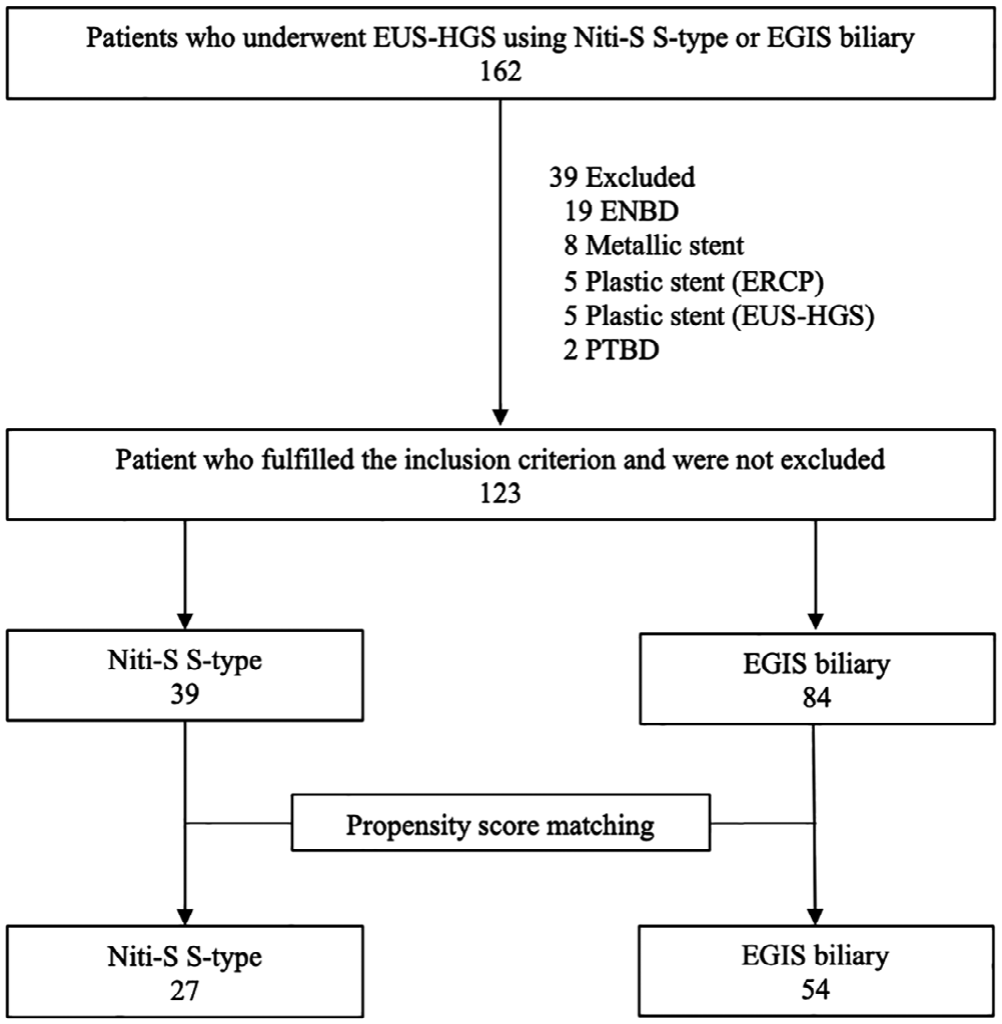
Study flowchart. EUS‐HGS, endoscopic ultrasound‐guided hepaticogastrostomy; ENBD, endoscopic nasobiliary drainage; ERCP, endoscopic retrograde cholangiopancreatography; PTBD, percutaneous transhepatic biliary drainage.

Among the 162 patients recruited from seven institutions, 123 (39 in the Niti‐S group and 84 in the EGIS group) were included after applying the exclusion criteria. After matching baseline characteristics, the matched cohorts comprised 27 and 54 patients in the Niti‐S and EGIS groups, respectively (Figure 2). In the pre‐matched cohort, differences were observed in history of cholecystectomy and use of AS; these variables were balanced after propensity score matching (Table 1). Data related to EUS‐HGS are presented in Table 2. HGS stent length differed between the two groups (*p* < 0.001), whereas puncture sites were similar. AS across the major duodenal papilla was performed in six and seven patients in the Niti‐S and EGIS groups, respectively. No patients underwent AS above the major duodenal papilla. Chemotherapy after EUS‐HGS was administered in seven (25.9%) and 21 patients (38.9%) in the Niti‐S and EGIS groups, respectively (*p* = 0.32).

**TABLE 1:** Baseline characteristics.

	Pre‐matched cohort				Matched cohort			
	Niti‐S (*n* = 39)	EGIS (*n* = 84)	*p*	SMD	Niti‐S (*n* = 27)	EGIS (*n* = 54)	*p*	SMD
Sex (male), *n* (%)	18 (46.2)	37 (44.0)	0.85	0.04	15	34	0.63	0.15
Age, years, median (IQR)	74 (62–81)	72 (65–77)	0.72	0.04	73 (63–80)	72 (65–80)	0.89	0.04
Performance status (0‐1), *n* (%)	21 (53.8)	46 (54.8)	1	0.03	15 (55.6)	33 (61.1)	0.64	0.11
History of cholecystectomy, *n* (%)	14 (35.9)	15 (17.9)	0.04	0.42	4 (14.8)	10 (18.5)	0.56	0.19
Gallstones, *n* (%)	5 (12.8)	13 (15.5)	0.6	0.14	4 (14.8)	5 (9.26)	0.72	0.13
Ascites, *n* (%)	16 (39)	23 (27.4)	0.15	0.29	9 (33.3)	20 (37)	0.81	0.08
Preprocedural cholangitis, *n* (%)	19 (48.7)	45 (53.6)	0.7	0.1	13 (48.1)	28 (51.9)	0.82	0.07
Total bilirubin level, mg/dL	5.6 (3.0–9.6)	4.9 (1.9–9.3)	0.7	0.03	5.4 (3.5–8.8)	4.8 (2.4–9.2)	0.64	0.003
Biliary obstruction site			0.18	0.27			1	0.04
Distal bile duct, *n* (%)	26 (66.7)	68 (81)			21 (77.8)	41 (75.9)		
Hilar bile duct, *n* (%)	13 (33.3)	18 (21.4)			6 (22.2)	13 (24.1)		
Primary site of cancer			0.92	0.29			0.95	0.12
Pancreas, *n* (%)	21 (53.8)	46 (54.8)			14 (51.9)	28 (51.9)		
Bile duct, *n* (%)	9 (23.1)	17 (20.2)			5 (18.5)	11 (20.4)		
Stomach, *n* (%)	4 (10.3)	12 (14.3)			4 (14.8)	9 (16.7)		
Others, *n* (%)	5 (12.8)	9 (10.7)			4 (14.8)	6 (11.1)		
Duodenal stent, *n* (%)	7 (17.9)	28 (33.3)	0.09	0.36	6 (22.2)	18 (33.3)	0.44	0.25
Antegrade stenting, *n* (%)	12 (30.8)	9 (10.7)	< 0.01	0.51	6 (22.2)	7 (13)	0.34	0.25

Abbreviatins: IQR, interquartile range; SMD, standardized mean difference.

**TABLE 2:** Procedural characteristics.

Variables	Subcategory	Niti‐S (*n* = 27)	EGIS (*n* = 54)	*p*‐value
Puncture site, *n* (%)	B2	11 (40.7)	17 (31.5)	0.46
	B3	16 (59.3)	37 (68.5)	
Use of dilation device, *n* (%)		25 (92.6)	54 (100)	0.1
Type of dilation devices, *n* (%)	Bougie	0	7 (12.9)	0.19
	Balloon	15 (64)	23 (42.6)	
	Cautery	8 (28)	10 (18.5)	
	Bougie and balloon	1 (4)	7 (12.9)	
	Bougie and cautery	1 (4)	1 (1.9)	
	Balloon and cautery	0	5 (9.3)	
	Bougie, balloon and cautery	0	1 (1.9)	
Stent diameter, *n* (%)	6 mm	1 (3.7)	0	0.33
	8 mm	26 (96.3)	54 (100)	
Stent length, *n* (%)	10 cm	13 (48.1)	0	<0.001
	12 cm	14 (51.9)	54 (100)	
Antegrade stenting site, *n* (%)	Across the papilla	6 (100)	7 (100)	1
	Above the papilla	0	0	
Procedure time, min, median (IQR)		28 (24–38)	30 (24–37)	0.47

Abbreviations: B2, intrahepatic bile duct in the segment 2; B3, intrahepatic bile duct in the segment 3; EUS‐HGS, EUS‐guided hepaticogastrostomy; IQR, interquartile range.

### 3.2 EUS‐HGS Outcomes

**FIGURE 3:**
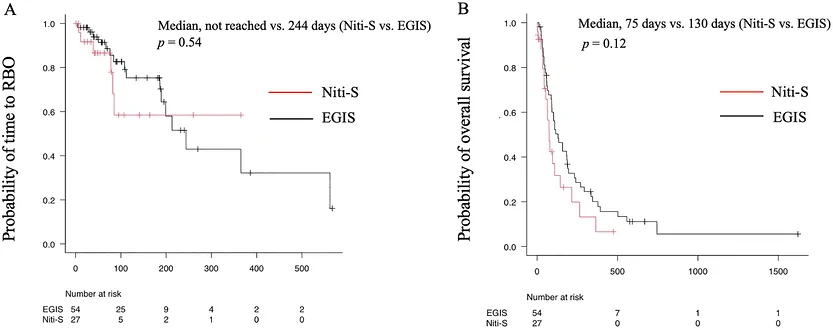
Comparison of the Niti‐S and EGIS groups with respect to TRBO and OS. (A) Kaplan–Meier curve showing cumulative TRBO in the Niti‐S and EGIS groups. No significant difference was observed (not reached vs. 244 days; *p* = 0.54, log‐rank test). (B) Kaplan–Meier curve showing cumulative OS in the Niti‐S and EGIS groups. No significant difference was observed (75 vs. 130 days; *p* = 0.12, log‐rank test).TRBO, time to recurrent biliary obstruction; OS, overall survival.

Clinical success rates were similar between groups: 88.9% (24/27) in the Niti‐S group and 98.1% (53/54) in the EGIS group (*p* = 0.11). TRBO and OS are shown in Figure 3. Similarity was observed in median TRBO (not reached in the Niti‐S group vs. 244 days in the EGIS group; *p* = 0.54) and median OS (75 vs. 130 days; *p* = 0.12). RBO occurred in six and 16 patients in the Niti‐S and EGIS groups, respectively, with no significant difference (*p* = 0.60) (Table 3). Patients who underwent AS during the index procedure did not develop RBO. Tumor invasion around the HGS stent occurred in one case in the Niti‐S group and in two cases in the EGIS group. In these patients, metastatic tumors or gallbladder cancer extended around the HGS, causing RBO. In one case in the EGIS group, kinking of the bile duct on the hepatic side of the stent resulted in RBO. To address the potential loss of statistical power after propensity score matching, Cox proportional hazards regression analysis was additionally performed using the entire cohort. Stent type was not independently associated with TRBO in the multivariable analysis (Table S1).

**TABLE 3:** Recurrent biliary obstruction.

	Niti‐S (*n* = 27)	EGIS (*n* = 54)	*p*‐value
Recurrent biliary obstruction, *n* (%)	6 (22.2)	16 (29.6)	0.60
Tumor invasion	1	2	
Hyperplasia	1	5	
Debris/stone	2	4	
Hemobilia	0	1	
Kink	0	1	
Unknown	2	3	

Non‐RBO AEs are summarized in Table 4. The early non‐RBO AE rate was significantly higher in the Niti‐S group (37% vs. 13%; *p* = 0.02). The primary causes of this difference were peritonitis and stent migration. Four cases of stent migration into the abdominal cavity were observed in the Niti‐S group (Table S2). Three of these involved stent migration 2–3 days after the procedure, resulting in peritonitis and/or severe sepsis. In these cases, the stents had not fully migrated into the abdominal cavity and were managed by placing an additional MS (*n* = 2) or plastic stent (*n* = 1) within the migrated HGS stent. In the remaining patient, AS was technically completed during the index procedure; however, the HGS stent was misplaced. Specifically, the hepatic end of the stent migrated into the hepatic parenchyma, whereas the gastric end remained in the stomach. The patient was treated by placing a nasobiliary drainage tube within the migrated HGS stent. Ten days after the procedure, cholangiography via the nasobiliary tube revealed no contrast leakage into the abdominal cavity, and the HGS stent was subsequently removed using a snare. One patient in the EGIS group experienced stent migration on postoperative day 2. The end of the stent remained in the stomach wall and was managed by placing an additional MS inside the stent (Table S2). Late non‐RBO AEs are summarized in Table 4. No significant difference was observed in late non‐RBO AEs. In the Niti‐S group, one stent moderately migrated into the esophagus and was pushed back into the stomach using a gastroscope. Additionally, a pseudoaneurysm that formed near the antegrade stent caused hematemesis, necessitating transarterial embolization (TAE) and exploratory laparotomy. However, the patient's condition deteriorated, resulting in death 2 days after surgery. The antegrade stent may have contributed to pseudoaneurysm formation. In the EGIS group, pseudoaneurysms, cholecystitis, and liver abscesses occurred; however, the index procedure was unlikely responsible for these AEs. A pseudoaneurysm developed around the pancreatic tumor and was treated with TAE. Cholecystitis likely resulted from cystic ductal invasion by the primary tumor.

**TABLE 4:** Non‐recurrent biliary obstruction (non‐RBO) adverse events.

	Niti‐S (*n* = 27)	EGIS (*n* = 54)	*p*‐value
Early non‐RBO adverse events			
Overall	10^†^ (37)	7 (13)	0.02
Mild/moderate/severe	3/4/3	3/4/0	0.45
Peritonitis	8	3	0.002
Mild/moderate/severe	3/2/3	2/1/0	
Stent migration	4	1	0.04
Mild/moderate/severe	0/1/3	0/1/0	
Cholangitis	0	1	1
Mild/moderate/severe	0/0/0	0/1/0	
Pancreatitis	0	1	1
Mild/moderate/severe	0/0/0	1/0/0	
Sepsis	2	0	0.11
Mild/moderate/severe	0/0/2	0/0/0	
Bleeding	1	0	0.33
Mild/moderate/severe	0/1/0	0/0/0	
Biloma	1	1	1
Mild/moderate/severe	0/1/0	0/1/0	
Late non‐RBO adverse events			
Overall	2 (7.4)	3 (5.6)	1
Mild/moderate/severe/fatal	0/1/0/1	1/2/0/0	1
Pseudoaneurysm	1	1	1
Mild/moderate/severe/fatal	0/0/0/1	0/1/0/0	
Stent migration into the esophagus	1	0	0.11
Mild/moderate/severe/fatal	0/1/0/0	0/0/0/0	
Liver abscess	0	1	1
Mild/moderate/severe/fatal	0/0/0/0	1/0/0/0	
Cholecystitis	0	1	1
Mild/moderate/severe/fatal	0/0/0/0	0/1/0/0	

Abbreviation: RBO, recurrent biliary obstruction.

^†^There were cases in which multiple adverse events occurred in a single patient.

## 4 Discussion

The outcomes of a high‐AF, cross‐type MS (Niti‐S) and a low‐AF, hook‐and‐cross‐type MS (EGIS) were compared using multicenter data adjusted for patient backgrounds. Early non‐RBO AEs occurred more frequently in the Niti‐S group, whereas the rates of clinical success, RBO, TRBO, OS, and late non‐RBO AEs were similar. EUS‐HGS using the Niti‐S biliary S‐type was retrospectively evaluated in 110 patients from two hospitals, reporting overall AE, peritonitis, and migration rates of 25%, 4%, and 0%, respectively [26]. These results differ from those observed in our Niti‐S group, where early non‐RBO AE, peritonitis, and migration rates were 37%, 29.6%, and 14.8%, respectively. However, this discrepancy requires further investigation. A key issue is whether stent migration occurs because of technical factors. Except for one case in the Niti‐S group, the gastric side of the stent migrated into the abdominal cavity a few days after EUS‐HGS, suggesting successful initial stent deployments. In the remaining case, the distal end of the stent migrated into the hepatic parenchyma, likely because of technical factors. Additionally, the EGIS biliary stent became available for EUS‐HGS in 2019, whereas the Niti‐S biliary S‐type has been available since 2012. Therefore, differences in endoscopist experience may have influenced outcomes. However, in this study, all data involved patients treated after 2019, across seven high‐volume hospitals, and the procedures were performed or supervised by experienced endoscopists. Thus, the higher non‐RBO AE rate in the Niti‐S group is unlikely attributable to nonproficiency.

**FIGURE 4:**
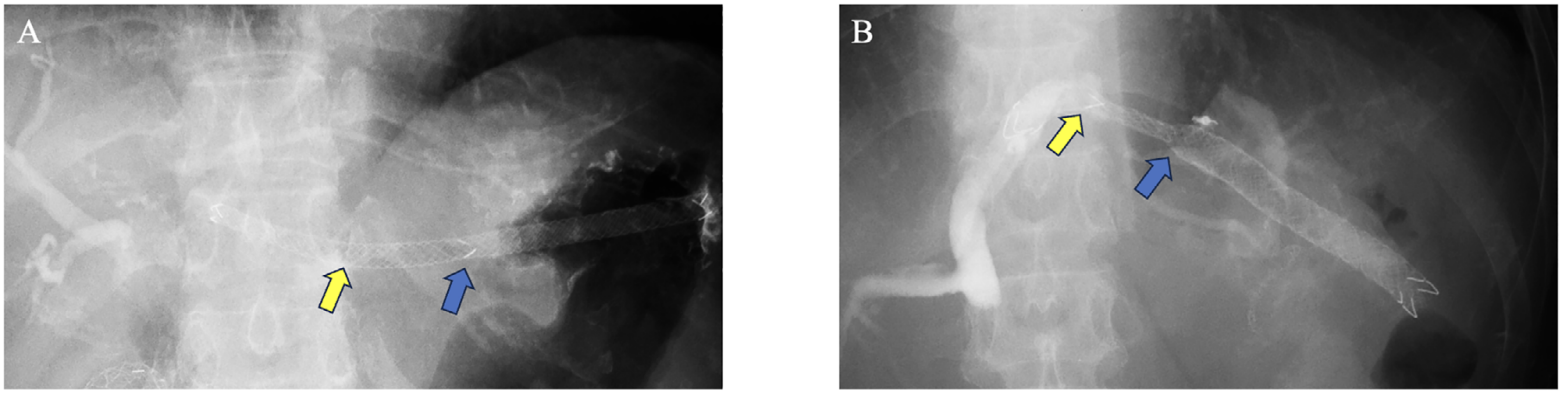
Fluoroscopic view of the hepaticogastrostomy stent immediately after placement between the intrahepatic bile duct and the stomach. The Niti‐S biliary S‐type tapers smoothly between the hepatic parenchyma and the bile duct or stomach (A), whereas the EGIS biliary stent forms a step (B). Yellow arrows indicate the boundary between the intrahepatic bile duct and the hepatic parenchyma, and blue arrows indicate the boundary between the hepatic parenchyma and the stomach.

A randomized controlled trial comparing two transpapillary MSs—the steel alloy MS (Wallstent, Boston Scientific) and the nitinol alloy MS (Wallflex; Boston Scientific, Marlborough, MA, USA)—demonstrated a higher stent migration rate with Wallstent (7% [14/200] vs. 2% [4/200]; *p* = 0.01) [27]. The authors suggested that the greater AF and rigidity of Wallstent contributed to its increased migration rate. This finding matches the higher rates of peritonitis and stent migration of the Niti‐S group in this study. We speculate that, in EUS‐HGS, MSs with high AF generate greater linear tension and may not conform adequately to the bile duct curvature. Furthermore, differences in expansion characteristics may influence early non‐RBO AE rates. Expansion behavior varies among laser‐cut, hook‐and‐cross‐type, and cross‐type stents. In general, cross‐type MSs taper smoothly into the hepatic parenchyma after deployment, whereas hook‐and‐cross‐type and laser‐cut MSs tend to create a step between the bile duct or stomach and the hepatic parenchyma (Figure 4). Cross‐type MSs may therefore be more susceptible to migration when external forces are applied. Tonozuka et al. evaluated the anchoring force of fully covered cross‐type and hook‐and‐cross‐type MSs using a benchtop EUS‐HGS model [28]. Anchoring force is defined as the resistance of the partially expanded distal portion of the MS to remain within the target organ against the pullback force of the sheath during deployment. Insufficient anchoring force may lead to stent migration. Although that study did not include the Niti‐S biliary S‐type, the EGIS biliary exhibited relatively high anchoring force compared with other cross‐type and hook‐and‐cross‐type MSs. Therefore, the hook‐and‐cross structure of the EGIS biliary stent may have anti‐migratory effects. However, one migration occurred in the EGIS group, indicating the need for further mechanical modifications, such as flared or flanged ends, on the gastric side. Factors other than AF influence MS properties. In transpapillary MS placement, radial force (RF) and the presence of a covering membrane may affect clinical outcomes [15, 16, 17, 18, 19]. RF refers to the outward expansion force of the stent. Laser‐cut MSs generally exhibit the highest RF, whereas cross‐type and hook‐and‐cross‐type MSs have relatively lower RF [20]. A benchtop study demonstrated similar RFs in the Niti‐S biliary S‐type and EGIS biliary stents [20]. Therefore, differences in RF are unlikely to have affected outcomes in this study. Although both MSs were partially covered, the length of the uncovered hepatic portion differed: 10 mm for Niti‐S and 20 mm for EGIS. Okamoto et al. reported that stent migration after EUS‐HGS only occurred with MSs having a short (5 mm) uncovered hepatic portion, compared with those having a longer (20 mm) uncovered portion [29]. In our study, the Niti‐S stent had a 10‐mm uncovered portion, which is longer than 5 mm but shorter than the 20‐mm portion of the EGIS stent, potentially influencing the results. Additionally, a 10‐cm stent was used in nearly half of the patients in the Niti‐S group, whereas only a 12‐cm stent was used in the EGIS group. Among the migrated Niti‐S stents, the length was 10 cm in three patients and 12 cm in one patient. Thus, stent length may also have affected the outcomes. However, in the previously mentioned retrospective study of the Niti‐S stent, a 10‐cm stent was used in 84% of patients, and no stents migrated [26]. This suggests that use of a 10‐cm stent alone does not fully explain the differences in AE rates. This study had several limitations. First, although propensity score matching reduces selection bias, it adjusts only for known confounders, and unmeasured confounders may remain. In addition, information regarding antithrombotic therapy was not consistently available across participating centers and was not included in the propensity score model. Second, despite the multicenter design, the sample size was relatively small, precluding definitive conclusions. In particular, the few patients in the Niti‐S group may have led to overestimation of the non‐RBO AE rate. Third, factors other than AF (e.g., lengths of stent and uncovered portion) differed between the groups; therefore, the isolated effect of AF cannot be definitively determined. Ideally, this question should be addressed in prospective studies comparing stents with different AFs but identical structural characteristics. In conclusion, the EGIS biliary stent exhibited fewer early non‐RBO AEs than the Niti‐S biliary S‐type stent after EUS‐HGS. Further studies are warranted to clarify the contributions of stent design characteristics to clinical outcomes.

## Author Contributions

**Masahiro Yamamura**: conceptualization, data curation, investigation, and Writing – original draft. **Junya Sato**: conceptualization, formal analysis, and investigation. **Hirotoshi Ishiwatari**: conceptualization, formal analysis, writing – original draft, and writing – review & editing. **Akifumi Notsu**: formal analysis. **Saburo Matsubara**, **Takeshi Okamoto**, **Michihiro Ono**, **Etsuji Ishida**, **Kohichi Takada**, **Kazuma Ishikawa**, **Naoki Sasahira**, **Kentaro Suda**, **Morito Ikeda**, and **Daichi Watanabe**: data curation and investigation. All authors reviewed and approved the final manuscript.

## Funding

The authors have nothing to report.

## Ethics Statement

**Approval of the research protocol by an Institutional Review Board**: This study was approved by the Institutional Review Board of Shizuoka Cancer Center (Approval No. T2024‐10‐2024‐1‐5).

## Consent

Written informed consent was obtained from all participants, or the requirement for informed consent was waived by the Institutional Review Board due to the retrospective nature of the study.

## Conflicts of Interest

The authors declare no conflicts of interest.

## Supporting information




**Supporting File 1**: deo270396‐sup‐0001‐TableS1‐S2.docx

## Data Availability

The data are not publicly available because of privacy or ethical restrictions but are available from the corresponding author upon reasonable request.
