# Supplementary material for: Comparison of Two Partially Covered Metal Stents for Endoscopic Ultrasound‐guided Hepaticogastrostomy: A Multi‐Center Retrospective Study
Source: DEN Open. 2026 Sep 10;7(1):e70396. doi: 10.1002/deo2.70396 (PMC13562807; doi:10.1002/deo2.70396)
Supplement: Supplementary file 1 [file DEO2-7-e70396-s001.docx]

Supplementary Table 1. Univariable and multivariable Cox proportional hazards regression analysis for factors associated with TRBO

|  | Univariable analysis | | | Multivariable analysis | |
| --- | --- | --- | --- | --- | --- |
|  | HR (95% CI) | *P*-value | | HR (95% CI) | *P*-value |
| Age ≧ 71 years | 0.63 (0.29-1.35) | | 0.23 |  |  |
| Performance status ≧ 2 | 1.12 (0.49-2.53) | | 0.79 |  |  |
| Male sex | 4.05 (1.52-10.8) | | 0.005 | 3.53 (1.29-9.63) | 0.014 |
| Pre-procedural cholangitis | 1.52 (0.69-3.36) | | 0.30 |  |  |
| Total bilirubin ≧ 5 mg/dL | 0.42 (0.18-0.99) | | 0.049 | 0.39 (0.16-0.96) | 0.041 |
| Chemotherapy after EUS-HGS | 0.83 (0.38-1.80) | | 0.63 |  |  |
| Niti-S biliary stent | 1.69 (0.67-4.23) | | 0.26 | 2.33 (0.72-7.61) | 0.16 |
| 12-cm stent | 0.28 (0.06-1.21) | | 0.09 | 0.61 (0.01-3.69) | 0.59 |
| Antegrade stenting | 0.22 (0.03-1.67) | | 0.14 | 0.26 (0.028-2.46) | 0.24 |

TRBO, time to recurrent biliary obstruction; CI, confidence interval; HR, hazard ratio; EUS-HGS, EUS-guided hepaticogastrostomy

Supplementary Table 2. Procedural details and management of patients with stent migration after EUS-HGS.

| Case | Stent type | Puncture site | Stent length | Antegrade stenting | Time to migration | Management |
| --- | --- | --- | --- | --- | --- | --- |
| 1 | Niti-S | B2 | 12cm | No | Day 2 | MS |
| 2 | Niti-S | B3 | 10cm | No | Day 2 | PS |
| 3 | Niti-S | B3 | 10cm | No | Day 3 | MS |
| 4 | Niti-S | B2 | 10cm | Yes | Day 0 | ENBD |
| 5 | EGIS | B3 | 12cm | No | Day 3 | MS |

B2, intrahepatic bile duct in the segment 2; B3, intrahepatic bile duct in the segment 3; EUS-HGS, EUS-guided hepaticogastrostomy; MS, metallic stent; PS, plastic stent; ENBD, endoscopic nasobiliary drainage
